# Supplementary material for: Inflammatory Signaling and DNA Damage Responses after Local Exposure to an Insoluble Radioactive Microparticle
Source: Cancers (Basel). 2022 Feb 18;14(4):1045. doi: 10.3390/cancers14041045 (PMC8869995; doi:10.3390/cancers14041045)
Supplement: Supplementary file 1 [file cancers-14-01045-s001.zip › cancers-1585930-supplementary.pdf]

Supplementary Material

Inflammatory Signaling and DNA Damage Responses after Local Exposure to an Insoluble Radioactive Microparticle

Yusuke Matsuya, Nobuyuki Hamada, Yoshie Yachi, Yukihiko Satou, Masayori Ishikawa, Hiroyuki Date, Tatsuhiko Sato

This supplementary material includes 6 figures, Figure S1: Geometry and dose profile of non-uniform exposure to 6MV-linac X-rays, Figure S2: Test for evaluating the effectiveness of the inhibitor, Figure S3 and S4: Spatial distribution of nuclear γ-H2AX foci for various inhibitor treatments for WI-38 cell line and HBEC3-KT cell line, respectively, Figure S5: NF-κB and COX-2 levels after non-uniform exposure, Figure S6: Dependency of irradiation area size on in- and out-of-field cell survival.

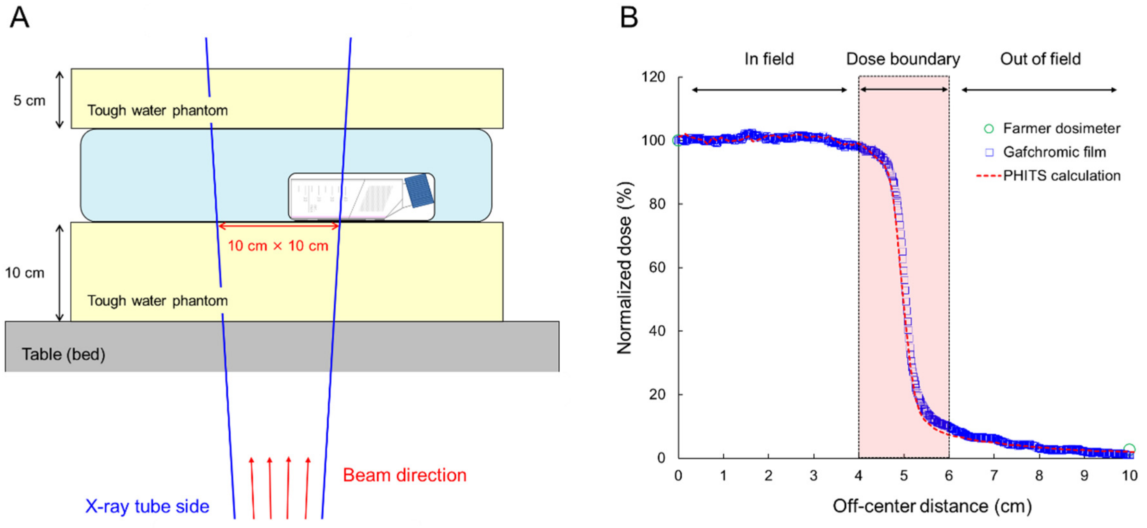

Figure S1. Geometry and dose profile of non-uniform exposure to 6 MV-linac X-rays: (A) Schematic illustration of geometry for irradiating 6 MV-linac X-rays. (B) Dose profile. As shown in Figure S1A, the 50% cells were exposed by the placement of cell culture container at the edge of the radiation field. The field size was 10 × 10 cm^2 and the depth was 10 cm. The dose profile was quantified by ionizing chamber, Gafchromic EBT3 film and the PHITS calculation using the phase-space file for Varian Clinac 600C 6MV photon (equivalent to Clinac 6EX). The out-of-field dose is 5%, on average, of in-field dose.

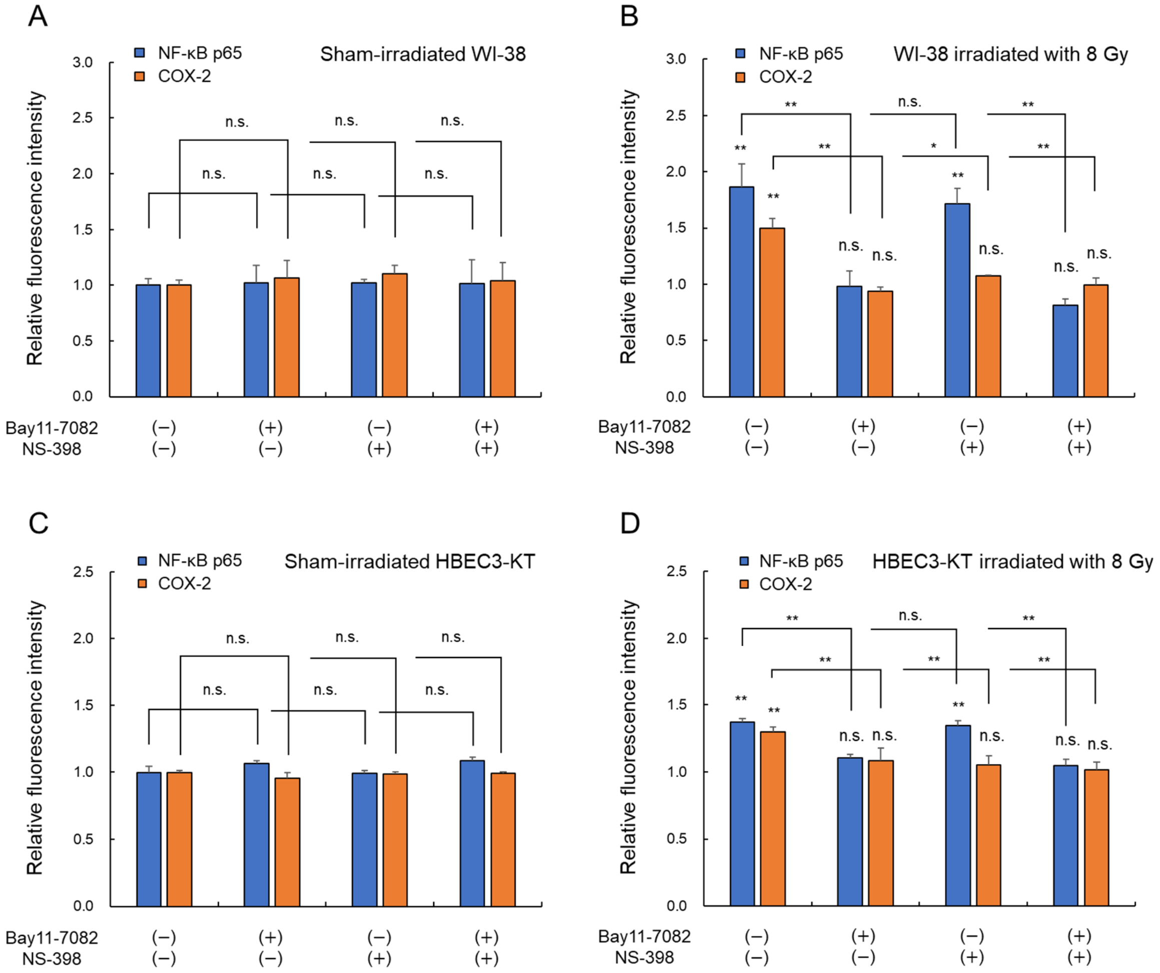

Figure S2. Test for evaluating the effectiveness of the inhibitor: (A) is for sham-irradiated WI-38 cells, (B) is for WI-38 cells irradiated with 8 Gy, (C) is for sham-irradiated HBEC3-KT, and (D) is for HBEC3-KT cells irradiated with 8 Gy. The inhibitors of NF-κB p65 and COX-2 used were 1 μM Bay-11-7082 (AG-CR1-0013-M010, Funakoshi) and 50 μM NS-398 (70590, Funakoshi), respectively. The irradiation was performed at room temperature with 150 kVp X-rays (1 mm Al filtration at 1.0 Gy/min) using an X-ray generator (MBR-1520R, Hitachi Medical Co., Tokyo, Japan). From Figure S2A and S2C, there was no significant difference among sham group and various inhibitor treatments. Bay-11-7082 inhibited both NF-κB p65 and COX-2 activations, and NS-398 reduced only COX-2 expression as shown in Figure S2B and S2D. Both treatments inhibited both NF-κB p65 and COX-2 activations in the same manner as NF-κB p65 treatment only. From these results, it was confirmed that NF-κB pathway upregulates COX-2 expression in inflammatory signaling pathways in WI-38 and HBEC3-KT cell lines. The symbols (*, **, n.s.) indicate the 5%, 1% significant difference and non-significant, respectively.

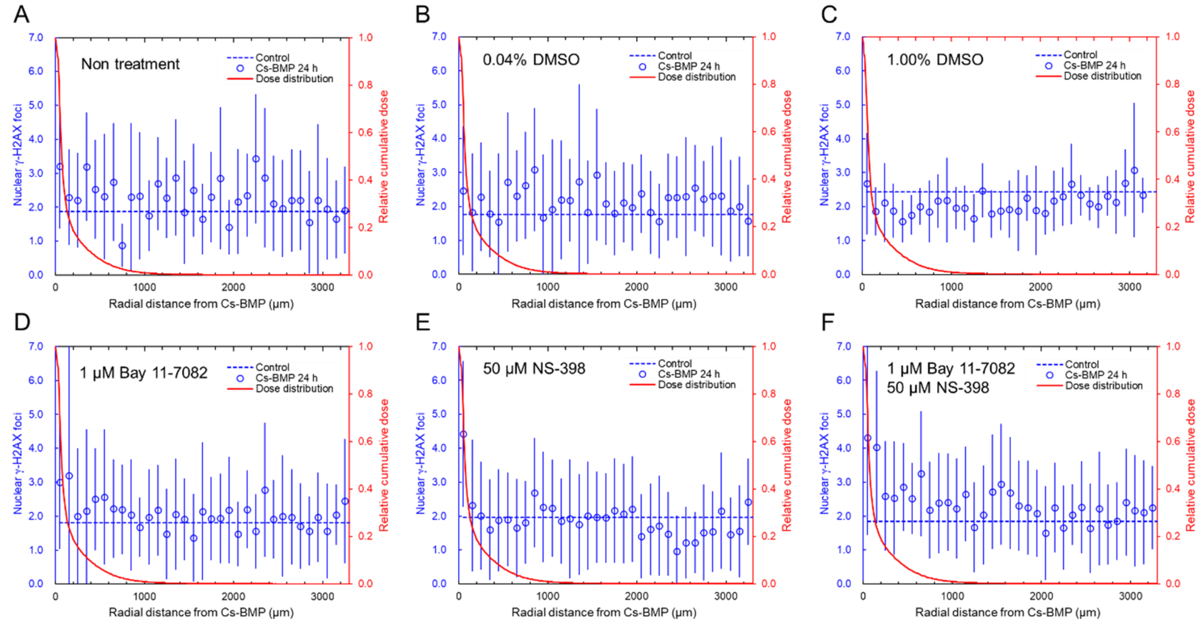

Figure S3. Spatial distribution of nuclear γ-H2AX foci for various inhibitor treatments for WI-38 cell line: (A) is for non-treatment, (B) is for 0.04% DMSO treatment, (C) is for 1.00% DMSO treatment, (D) is for 1 μM Bay 11-7082 treatment, (E) is for 50 μM NS-398 treatment, and (F) is for both treatments with 1 μM Bay 11-7082 and 50 μM NS-398. Blue dotted line is the non-irradiated level. Red solid line is the dose profile.

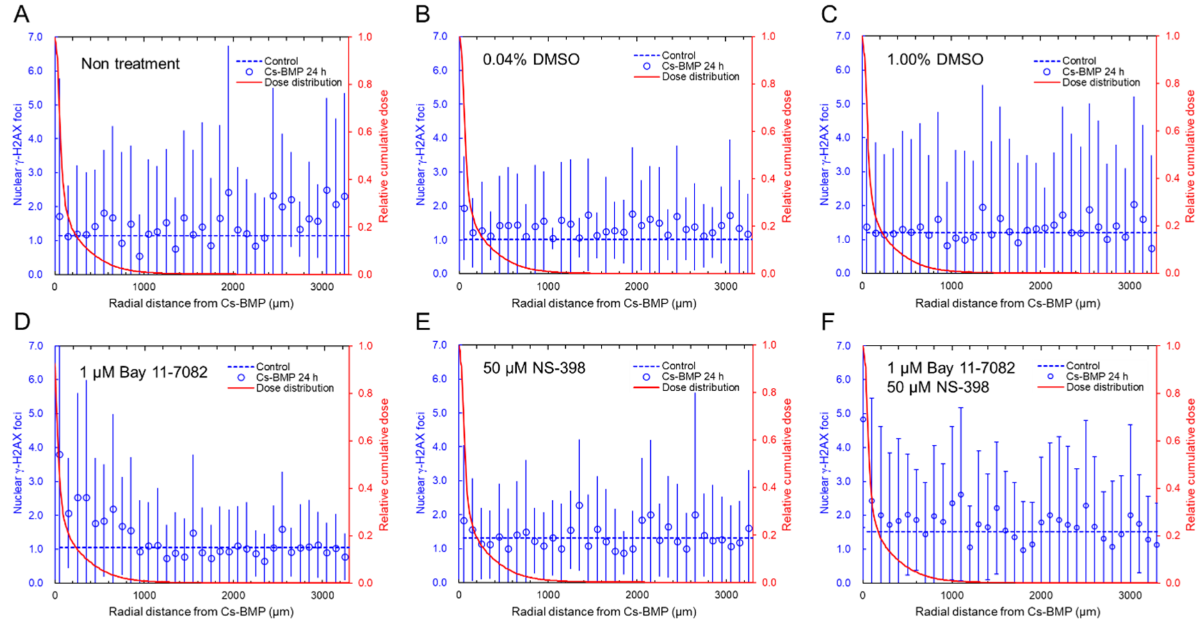

Figure S4. Spatial distribution of nuclear γ-H2AX foci for various inhibitor treatments for HBEC3-KT cell line: (A) is for non-treatment, (B) is for 0.04% DMSO treatment, (C) is for 1.00% DMSO treatment, (D) is for 1 μM Bay 11-7082 treatment, (E) is for 50 μM NS-398 treatment, and (F) is for both treatments with 1 μM Bay 11-7082 and 50 μM NS-398. Blue dotted line is the non-irradiated level. Red solid line is the dose profile.

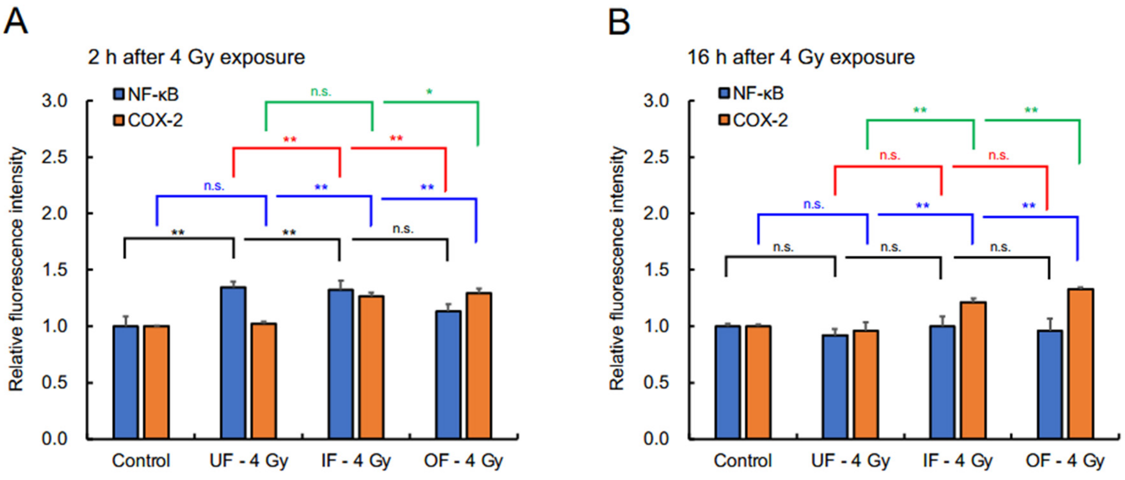

Figure S5. NF-κB and COX-2 levels after non-uniform exposure: (A) is the relative fluorescence to sham-irradiated WI-38 cells at 2 h post-irradiation, (B) is the relative value to sham-irradiated WI-38 cells at 16 h post-irradiation. In this non-uniform exposure, the dose was delivered to 50% of the area of culture dish containing the cells, and the in-field dose was 4 Gy. The irradiation was performed at room temperature with 150 kVp X-rays (1 mm Al filtration at 1.0 Gy/min) using an x-ray generator (MBR-1520R, Hitachi Medical Co., Tokyo, Japan). The significant activations of NF-κB and COX-2 in out-of-field cells were detected at 2 h after non-uniformexposure to 4 Gy (figure S5A) as bystander responses. Meanwhile, the persistent COX-2 expression was also detected at 16 h after the exposure (figure S5B). The symbols (*, **, n.s.) indicate the 5%, 1% significant difference and non-significant, respectively.

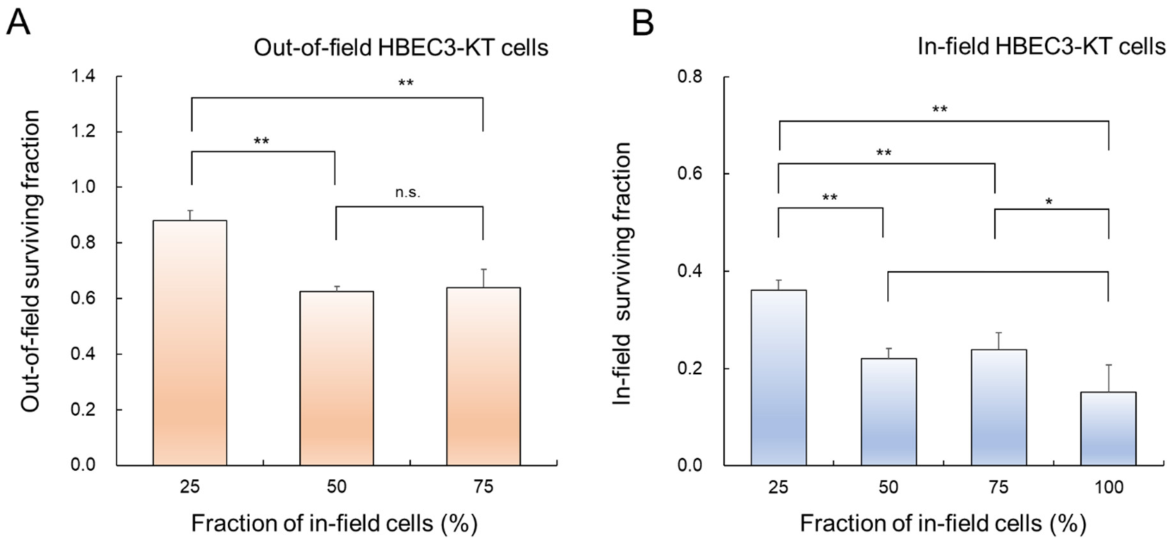

Figure S6. Dependency of irradiation area size on in- and out-oh-field cell survival: (A) is out-of-field survival of HBEC3-KT cells, (B) is in-field survival of HBEC3-KT cells. The partial irradiation was performed and the in-field dose was 4 Gy. The irradiation was performed at room temperature with 6-MV linac X-rays (Clinac 6EX, Varian). The geometry and dose profile are described in Figure S1. As shown in Figure S6A, the bystander effects (reduced clonogenicity) from in-field cells to out-of-field seems to be saturated for larger fraction of in-field cells than 50%. Meanwhile, the protective effects (reduced cell killing) are maximized in case of the smallest fraction of in-field cells of 25%. The symbols (*, **, n.s.) indicate the 5%, 1% significant difference and non-significant, respectively.
